# Effectiveness of exercise interventions on gross motor skills in children with autism spectrum disorder: a systematic review and meta-analysis

**DOI:** 10.3389/fpsyt.2026.1745638

**Published:** 2026-02-26

**Authors:** Mengyao Feng, Yaoqi Hou, Shenning Zhou, Xiangqin Song

**Affiliations:** School of Physical Education and Sports, Beijing Normal University, Beijing, China

**Keywords:** autism spectrum disorder, exercise intervention, gross motor skills, meta-analysis, randomized controlled trial

## Abstract

**Objective:**

To systematically evaluate the effectiveness of different exercise interventions on gross motor skills in children with Autism Spectrum Disorder (ASD) and to compare the effects of different exercise types and intervention dosages through subgroup analysis.

**Methods:**

We systematically searched randomized controlled trials (RCTs) and non-randomized controlled trials (NRCTs) investigating the effects of exercise interventions on gross motor skills in children with ASD across PubMed, Web of Science, EBSCO, ProQuest, CNKI, and Wanfang Data databases from their inception until September 20, 2025. Two researchers independently performed literature screening, data extraction, and quality assessment of the included studies using the Cochrane Risk of Bias tool. Meta-analysis was conducted using RevMan 5.4 software with a random-effects model, and effect sizes were expressed as standardized mean difference (SMD) with 95% confidence intervals (CI).

**Results:**

A total of 16 studies involving 493 children with ASD were included. Meta-analysis results indicated that exercise interventions significantly improved balance skills (SMD = 0.85, 95% CI [0.54, 1.16]), locomotor skills (SMD = 0.81, 95% CI [0.51, 1.11]), and object control skills (SMD = 0.86, 95% CI [0.65, 1.07]) in children with ASD, with all effect sizes being large (all P < 0.00001). Subgroup analysis showed that Land-based sports significantly improved all three skill domains (all P < 0.00001), whereas Aquatic sports and technically-assisted sports mostly failed to reach statistical significance. Regarding intervention dosage, medium-duration programs (total duration ≤1440 minutes) significantly improved all three skill domains (all P < 0.0001), while high-duration programs (>1440 minutes) only showed a significant effect on object control skills (SMD = 0.75, P = 0.003).

**Conclusion:**

Exercise interventions, particularly structured Land-based sports, are effective methods for improving gross motor skills in children with ASD. A medium-dosage regimen with a total intervention duration of ≤1440 minutes (approximately 8–12 weeks) appears to be the key window for optimal benefits. Prioritizing this regimen in clinical rehabilitation and educational practice is recommended.

**Systematic Review Registration:**

https://www.crd.york.ac.uk/PROSPERO/, identifier CRD420251185572.

## 1 Introduction

Autism Spectrum Disorder (ASD) is a multifaceted neurodevelopmental disorder. Children with ASD commonly experience pervasive difficulties in social communication, including deficits in social-emotional reciprocity, social interaction, and the development, maintenance, and understanding of relationships. They also exhibit restricted, repetitive patterns of behavior, interests, or activities in daily life (1, 2). Functional communication impairments present in nearly 71-80% of individuals with autism may be linked to cognitive-behavioral issues, which is a primary reason for the severe impairment of social function in autism (3). Although motor skills are not considered a core diagnostic feature of ASD, apart from social deficits, up to 83% of children with ASD have difficulty performing age-appropriate motor skills (4, 5). Motor skill deficits or delays can be observed throughout childhood in children with ASD, primarily including coordination disorders, clumsiness, slower movement times, poor balance, motor impairments, and impaired gestural performance (6, 7). Balance and ball skills are common and significant deficits in children with ASD (8). Compared to their neurotypical peers, children with ASD engage in less physical activity, with motor delays/defects and gross motor limitations considered the most significant barriers to their participation in physical activities with peers (9, 10).

Motor development is typically categorized into gross motor skills (involving movements of large muscle groups) and fine motor skills (involving coordination of small muscle groups) (11, 12). For the research and practical assessment of gross motor skills, to more precisely identify and intervene in specific ability deficits, the academic field often employs a three-dimensional classification framework based on the Test of Gross Motor Development (TGMD), namely: balance skills (e.g., static and dynamic stability), locomotor skills (e.g., running, jumping), and object control skills (e.g., throwing, catching) (13). These three dimensions collectively form the core of children’s fundamental movement competence and are closely related to daily activities, play participation, and the development of higher-order sports skills. Therefore, this study adopts this tripartite framework as the analytical structure, aiming to systematically evaluate the effects of exercise interventions on these three specific dimensions in children with ASD, thereby providing more instructive evidence-based guidance than an overall “gross motor” score.

Currently, improving the core symptoms and gross motor abilities of children with ASD primarily relies on two main approaches: pharmacological treatment and conventional behavioral strategies (14, 15). However, these two methods face limitations in widespread implementation due to factors such as time cost, financial cost, and dependence on professional expertise (16). As a non-pharmacological treatment method, exercise intervention demonstrates distinct advantages in cost-effectiveness, ease of implementation, and safety (17, 18). Consequently, in recent years, it has been widely regarded as an effective and feasible intervention, capable of not only improving the core symptoms of children with ASD but also positively promoting their gross motor skills, particularly balance, object control, and locomotor abilities (19–21).

Meta-analysis (MA) allows for the integration of various interventions and the comparison of their effectiveness based on unified outcome measures. It has proven effective in synthesizing all available evidence even when direct comparative data are limited. Although exercise has been demonstrated to effectively improve gross motor skills in children with ASD, previous studies suffer from issues such as high heterogeneity in intervention types, small sample sizes, and inconsistent conclusions. There is currently a lack of systematic reviews and MAs that finely categorize by different exercise types (e.g., land-based, aquatic, technology-assisted) and intervention doses (e.g., total duration). While some studies have used MA to explore the overall impact of exercise on children with ASD, constrained by the aforementioned heterogeneity and other factors, they have not compared the improvement effects of different exercise intervention types and doses on the various dimensions of gross motor skills in children with ASD. Therefore, the purpose of this study is to systematically compare the effectiveness of different exercise types and intervention doses on the gross motor skills of children with ASD through subgroup analysis, aiming to provide more refined evidence-based guidance for clinical practice (22).

## 2 Methods

This review and meta-analysis adhered to the 2020 updated Preferred Reporting Items for Systematic Reviews and Meta-Analyses (PRISMA) statement, and the PRISMA checklist is provided below. (Registration number: CRD420251185572).

### 2.1 Literature search strategy

A comprehensive search was conducted on the topic “effectiveness of exercise interventions on improving gross motor skills in children with Autism Spectrum Disorder” to identify all studies reporting changes in gross motor skills following exercise interventions in autistic children. Six databases were systematically searched: China National Knowledge Infrastructure (CNKI), Wanfang Data, PubMed, ProQuest, Web of Science, and EBSCO, from their inception to September 20, 2025. The search strategy employed a combination of keywords and Medical Subject Headings (MeSH) terms, constructed around four core concepts: Autism Spectrum Disorder (autism, ASD, etc.), Exercise Intervention (physical activity, sports, etc.), Gross Motor (motor skills, gross motor skills, etc.), and Children (child, preschool, etc.). Synonyms within each concept group were connected using the Boolean operator “OR,” and different concept groups were combined using “AND.” To ensure complete transparency and reproducibility, the reproducible search syntax executed across all six databases is provided as a **Supplementary File** (**Table 1**). Below is an example of the core keyword framework used to construct the search strategy. A detailed description of the search strategy is provided in the Appendix.

**Table 1:** The core concepts and keyword framework of the search strategy.

Core concept	English key words/search terms
Person	“Autism Spectrum Disorder”, autistic, ASD, asperger*
Intervention	exercise, “physical activity”, sport*, “motor intervention”
Outcome	“motor skill*”, “gross motor”, locomotor, “object control”
Range	child, preschool, kid, toddler*

### 2.2 Inclusion and exclusion criteria

The Zotero software was used to screen the retrieved literature. Inclusion criteria were determined based on the PICOS principle. Participants (P) were children aged 3–12 years diagnosed with Autism Spectrum Disorder. Interventions (I) included land-based exercise training, aquatic exercise training, comprehensive exercise, and technology-assisted interventions (e.g., VR technology). Comparisons (C) were blank controls, routine exercise, or different exercise interventions from the experimental group. Outcome measures (O) were scores from relevant assessment tools, such as the gross motor sections of HAAR, BOTMP, PDMS-2, TGMD-2/3, BOT-2, MABC-2, etc. Study designs (S) included randomized controlled trials and non-randomized controlled trials.

Exclusion criteria were as follows: ① Participants were not diagnosed with ASD or had other severe neurological/musculoskeletal comorbidities; ② Interventions were non-exercise-based, or the comparison between experimental and control groups did not reflect the value of exercise intervention; ③ Non-Chinese or non-English literature; ④ Literature for which the full text was unavailable, had incomplete data, or was duplicate publications; ⑤ Studies that did not report complete data for the gross motor development assessment tools relevant to this study; ⑥ Non-controlled trials.

### 2.3 Outcome measures and assessment tools

The primary outcome of this study was the change in standardized assessment scores for gross motor skills in children with ASD. The included studies utilized various internationally recognized assessment tools, mainly including: (1) Comprehensive motor development tests: Gross motor sections of the *Peabody Developmental Motor Scales-Second Edition (PDMS-2)*, and the *Bruininks-Oseretsky Test of Motor Proficiency (Second Edition/Revised) (BOT-2/BOTMP)*. These tools provide composite or subscale scores for dimensions such as balance, locomotion, and object control. (2) Fundamental motor skill-specific tests: *Test of Gross Motor Development-Second/Third Edition (TGMD-2/TGMD-3)*, specifically designed to assess locomotor and object control skills. (3) Other tools: Hong Kong Assessment of Attention for Autistic Children (HAAR), etc. Despite the diversity of tools, their core focuses revolve around the three major gross motor skill domains: balance, locomotion, and object control. For data synthesis, we extracted post-test group means, standard deviations, and sample sizes for these three dimensions or the most closely related dimensions from each study. If a study only reported total scores, it was included in the overall effect analysis but not in the skill-dimension subgroup analyses.

### 2.4 Literature screening and data extraction

Studies were initially screened by title and abstract, followed by assessment of full-text eligibility. Discrepancies were resolved by consulting a third expert. Information extracted from the studies included participant characteristics (age, gender, country, diagnostic method, sample size), intervention components (intervention type, intensity, duration, frequency, etc.), outcome measures, and results (mean differences in gross motor changes in children with ASD after exercise intervention).

### 2.5 Methodological quality assessment

Two researchers independently assessed the methodological quality of the included randomized controlled trials and quasi-experimental studies using the Cochrane Risk of Bias tool. The assessment focused on: random sequence generation, allocation concealment, blinding of participants and personnel, completeness of outcome data, selective reporting, and other potential biases. Discrepancies in quality ratings were resolved through discussion with a third researcher to reach consensus (23).

### 2.6 Data synthesis and analysis

This study used Review Manager (RevMan) 5.4 software, recommended by the Cochrane Collaboration, for data synthesis and analysis.

#### 2.6.1 Effect size and statistical model

For continuous outcomes, the included studies used different assessment tools (e.g., HAAR, BOTMP, PDMS-2, TGMD-2/3, BOT-2, ADS-3), resulting in differences in scales and units of measurement. To eliminate these differences and enable the combination of results from different tools, this study employed the standardized mean difference (SMD) as the pooled effect size. In RevMan 5.4, the SMD is calculated based on Hedges’ g to correct for small-sample bias. The SMD is standardized by the internal standard deviation of the effect size, reflecting the degree of intervention effect relative to within-group variation. Fully aware of the potential heterogeneity arising from different tools and study variations, this study used a random-effects model for pooling to more conservatively estimate the pooled effect size and its confidence interval. In result interpretation, a positive SMD indicates that the exercise intervention group performed better on the motor function indicator than the control group. According to Cohen’s conventional interpretation standards, an SMD ≈ 0.2 is considered a small effect, 0.5 a medium effect, and 0.8 a large effect.

#### 2.6.2 Heterogeneity assessment

The I² statistic was used to assess heterogeneity among studies. In this study, I² values were interpreted as follows: 25%, 50%, and 75% represented low, moderate, and high heterogeneity, respectively. If I² > 50%, it was considered that non-negligible heterogeneity existed, and the analysis of study characteristics would be conducted to explore its potential sources.

#### 2.6.3 Subgroup analysis and sensitivity analysis

To explore sources of heterogeneity and address specific research questions, the following analysis plan was pre-specified:

##### 2.6.3.1 Subgroup analysis

Subgroup Analysis: This study conducted subgroup analyses based on the core mechanisms of action of exercise interventions and total dose to deeply explore their potential impact on the three sub-dimensions of gross motor skills in children with ASD: balance, object control, and locomotor skills. Specifically, interventions were first categorized into three subgroups: (1) Land-based sports: Traditional exercise forms conducted in terrestrial environments focusing on neuromuscular control and basic physical fitness training (24); (2) Aquatic sports: Exercises utilizing the buoyancy and resistance properties of water to reduce joint load and provide unique physiological stimulation (25); (3) Technology-assisted interventions:Technology-assisted intervention refers to a new training approach that enhances sensory input and motor learning by leveraging advanced technologies such as virtual reality (26). To further investigate the “dose-effect” relationship of the intervention, this study, based on the distribution of intervention durations in most included studies (8–12 weeks, 2–3 times per week, 30–60 minutes each time) and common cycles in clinical practice, pre-defined the cut-off points for the total intervention duration for subgroup analysis. Specifically, the total intervention duration of ≤1440 minutes (approximately 8–12 weeks) was defined as the moderate-dose group; and >1440 minutes was defined as the high-dose group (27). The setting of this threshold aims to explore the intervention duration window that may produce the best benefits based on the existing evidence distribution. Ultimately, the differences in effect sizes resulting from different intervention types and durations will be evaluated through inter-group difference tests to determine if they are statistically significant.

##### 2.6.3.2 Sensitivity analysis

Sensitivity analysis: To test the robustness of the pooled results, sensitivity analyses were conducted as follows: (1) Excluding studies with an overall “high risk” of bias; (2) Excluding studies with imputed data; (3) Analyzing only RCTs, excluding single-group pre-post studies; (4) Changing the meta-analysis statistical model (fixed-effect vs. random-effects model).

##### 2.6.3.3 Publication bias assessment

If a meta-analysis included ≥ 10 studies, publication bias was assessed by generating funnel plots and visually inspecting them.

### 2.7 Literature screening process

**Figure 1:**
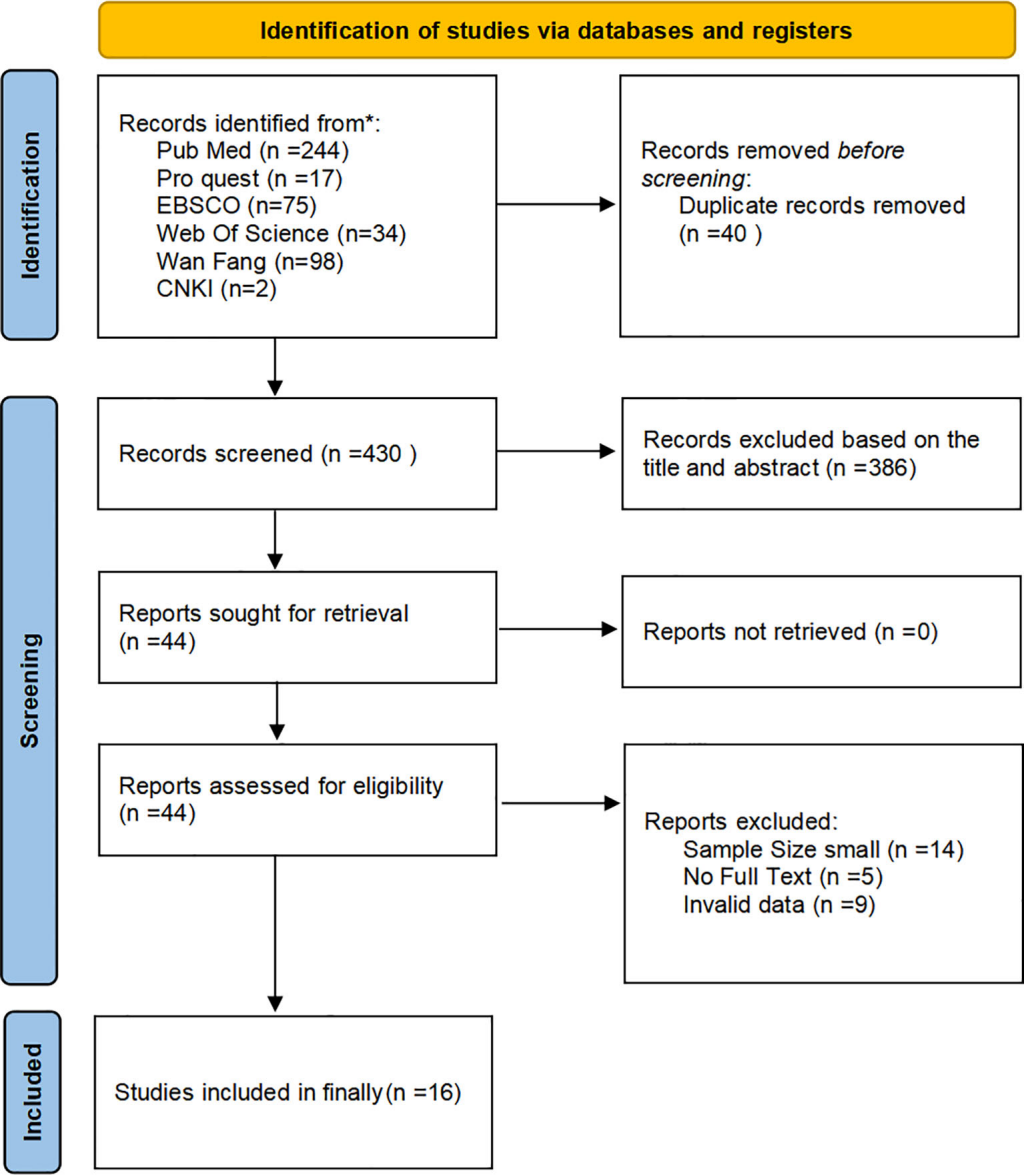
Literature screening flowchart (PRISMA).

**Figure 1** illustrates the study selection flow. After searching the six databases, a total of 450 records were identified. Following the removal of duplicates, 430 unique records remained. After assessing full-text articles for eligibility, 414 were excluded for the following reasons: excluded by title/abstract (n=386), invalid data (n=9), insufficient sample size (n=14), full text unavailable (n=5). Ultimately, 16 studies were included in the systematic review.

## 3 Results

### 3.1 Characteristics of included studies

**Table 2** presents the basic characteristics of the 16 included studies (total N = 493 children aged 3-12) conducted in multiple countries/regions from 2014 to 2025. All studies used standardized diagnostic tools. Intervention types included Land-based sports, Aquatic sports, and technology-assisted training. Specific doses and designs are detailed in **Table 2**.

**Table 2:** Basic characteristics of included studies.

Study	Country	Diagnostic	Intervention	Study design	E/C	Total minute	Week	Frequency	Single duration	Outcome indicator
(28)	Canada	DSM-V	Land-based sports	WLCD	5/4	1440	12	2	60	PDMS-2
(29)	Columbia	DSM-V	Land-based sports	RCT	10/10	1440	8	3	60	ADS-3
(30)	America	DSM-V	Aquatic sports	CCD	71/20	960	8	2	60	BOT-2
(31)	China	DSM-V、ADI-、C-PEP3	Land-based sports	RCT	8/10	1800	10	3	60	TGMD-3
(32)	Republic of Lithuania	DSM-V	Technical assistance sports	RCT	10/10	450	5	3	30	BOTMP
(33)	America	DSM-V	Aquatic sports	WLCD	7/5	1120	14	2	40	YMCA、M-PEDI
(34)	China	DSM-V	Land-based sports	RCT	6/6	1440	8	3	60	TGMD-2
(35)	Iran	GARS-2、WISC-4	Land-based sports	RCT	8/8	1440	8	3	60	GARS-2
(36)	Turkey	DSM-V、GARS-2-TV	Land-based sports	RCT	17/17	1440	12	2	60	PMDS-2
(37)	America	DSM-IV、ADOS-2	Land-based sports	QED	11/9	9600	8	5	240	TGMD-2
(38)	Korea	DSM-V	Technical assistance sports	RCT	27/25	1080	12	2	30	TGMD-3
(39)	Iran	DSM-IV-TR、GARS-2	Land-based sports	QED	14/14	1440	12	3	40	BOTMP
(40)	Taiwan, China	DSM-IV	Aquatic sports	COD	8/8	1680	12	2	70	HAAR
(41)	Turkey	DSM-V	Land-based sports	MMR	13/18	1440	12	2	60	BOT-2
(42)	Morocco	DSM-V	Land-based sports	QED	7/7	2025	15	3	45	UQAC-UQAM
(43)	China	DSM-V	Land-based sports	RCT	50/50	1200	10	2	60	ABC

DSM-IV, Diagnostic and Statistical Manual of Mental Disorders, Fourth Edition; DSM-IV-TR: Diagnostic and Statistical Manual of Mental Disorders, Fourth Edition, Text Revision; DSM-V, Diagnostic and Statistical Manual of Mental Disorders, Fifth Edition; ADI-R, Autism Diagnostic Interview-Revised; C-PEP3, Psychoeducational Profile-Third Edition (Chinese Version); GARS-2, Gilliam Autism Rating Scale-Second Edition; GARS-2-TV, Gilliam Autism Rating Scale-Second Edition (Turkish Version); WISC-4, Wechsler Intelligence Scale for Children-Fourth Edition; ADOS-2, Autism Diagnostic Observation Schedule, Second Edition; RCT, Randomized Controlled Trial; WLCD, Wait-List Controlled Design; CCD, Cross-Over Controlled Design; QED, Quasi-Experimental Design; COD, Cohort Controlled Design; MMR, Mixed Methods Research; PDMS-2, Peabody Developmental Motor Scales-Second Edition; ADS-3, Adaptive Behavior Scale-Third Edition; BOT-2, Bruininks-Oseretsky Test of Motor Proficiency-Second Edition; TGMD-3, Test of Gross Motor Development-Third Edition; BOTMP, Bruininks-Oseretsky Test of Motor Proficiency (Revised); YMCA, Young Men’s Christian Association Children’s Fitness Test; M-PEDI, Motor Function Evaluation Scale for Children; TGMD-2, Test of Gross Motor Development-Second Edition; PMDS-2, Peabody Motor Development Scale-Second Edition; HAAR, Hong Kong Assessment of Attention for Autistic Children; ABC, Autism Behavior Checklist.

### 3.2 Risk of bias assessment

**Figure 2:**
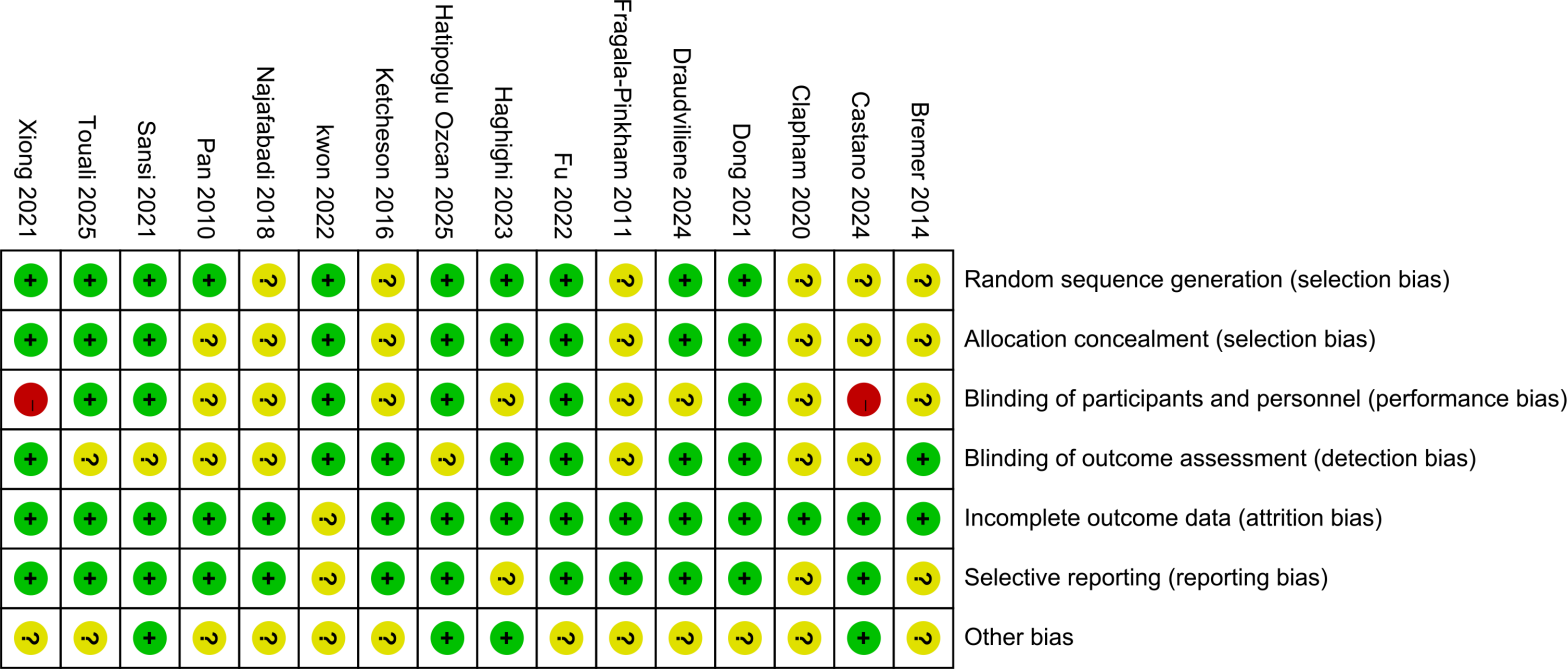
Risk of bias graph for included studies.

**Figure 3:**
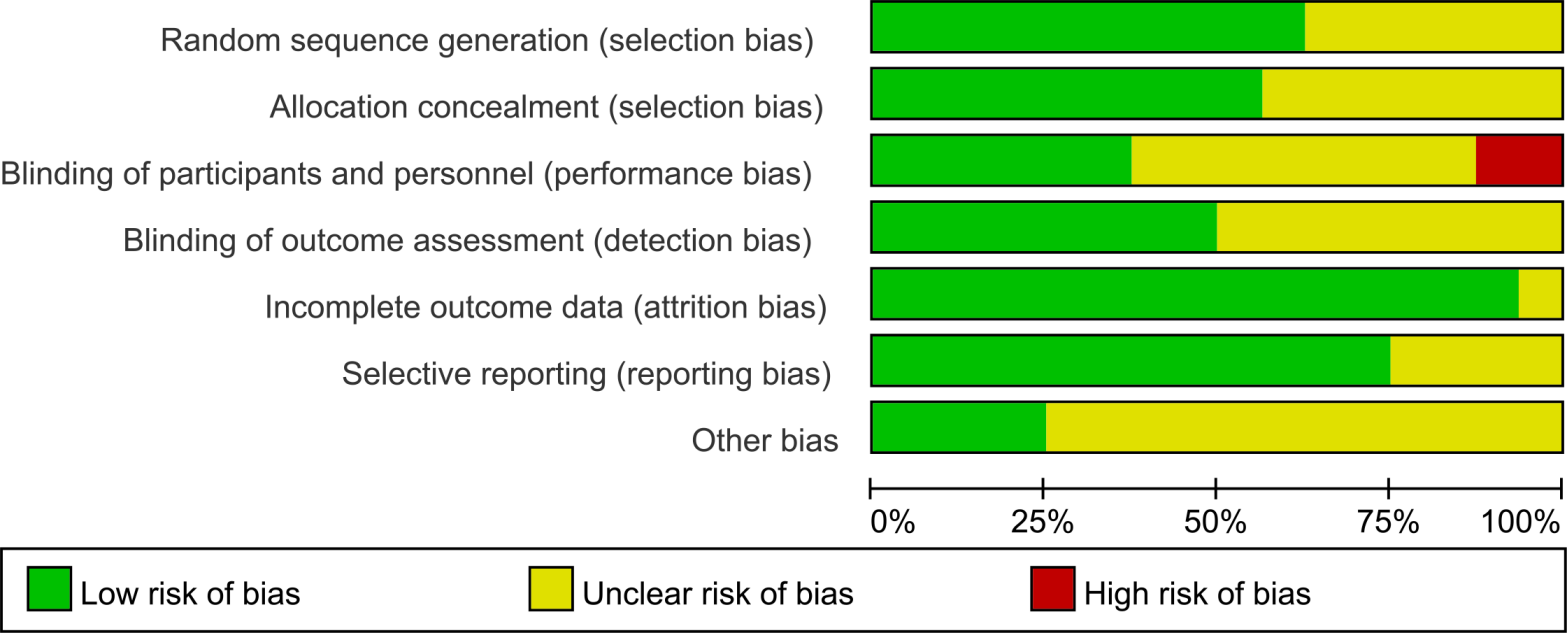
Risk of bias summary for included studies.

**Figure 2** illustrates the risk of bias across the included studies. Across the five domains, some concerns were noted in the 16 studies, primarily related to selection bias and performance bias (**Figure 3**). Regarding randomization process, detection bias, attrition bias, and reporting bias, the included studies demonstrated low risk of bias.

### 3.3 Meta-analysis

#### 3.3.1 Meta-analysis results

**Figure 4:**
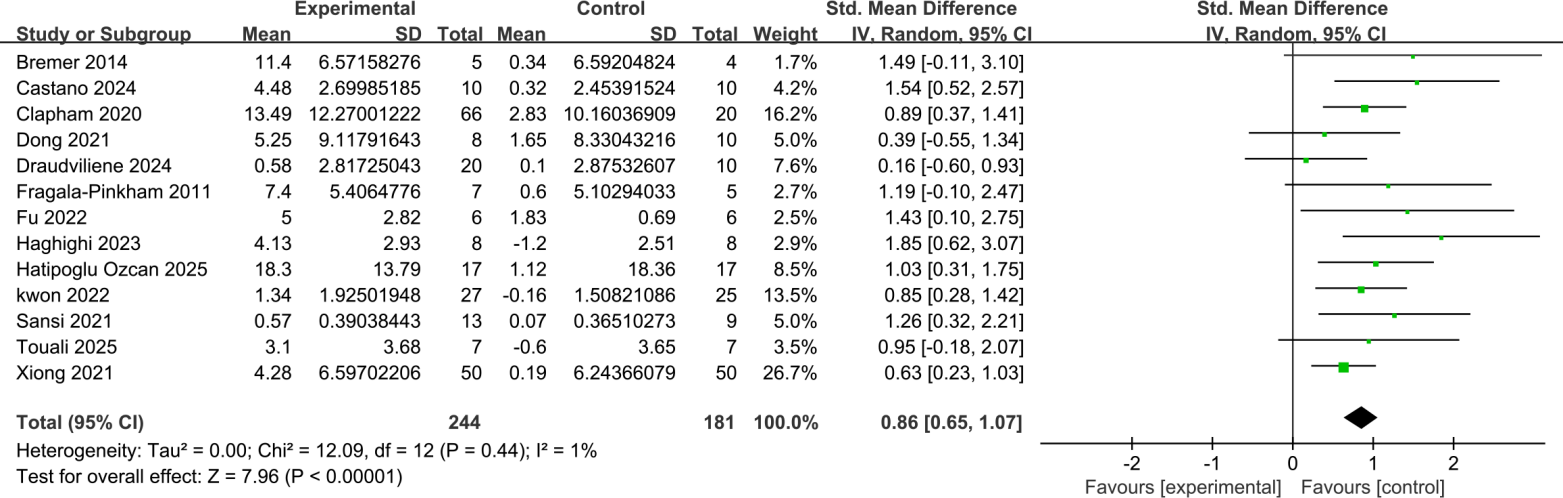
Forest plot of the effect of exercise intervention on control skills in children with ASD (random effects model).

This study comprehensively evaluated the effects of exercise interventions on balance, object control, and locomotor skills in children with ASD using a random-effects model. Specific results are as follows:

For object control skills, 13 studies (n=425) reported the effects of exercise interventions. Meta-analysis results (see **Figure 4**) showed a statistically significant pooled effect size (SMD = 0.86, 95% CI [0.65, 1.07], Z = 7.96, P < 0.001). This indicates that exercise interventions significantly improved object control skills in children with ASD, with a large effect size (SMD > 0.8). Heterogeneity among studies was very low (I² = 1%, P = 0.44), further supporting the reliability of this result.

**Figure 5:**
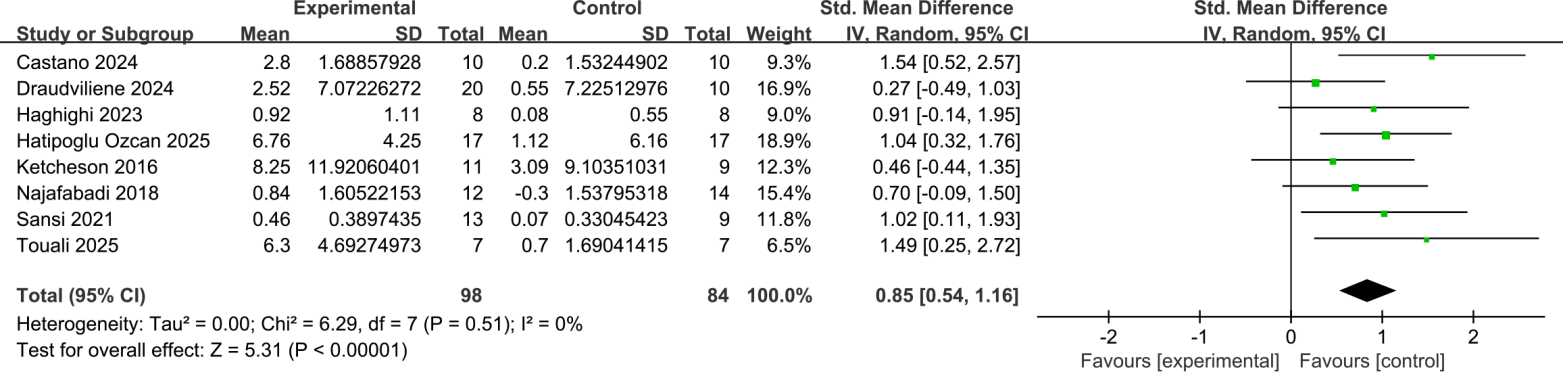
Forest plot of the effects of exercise intervention on balance skills in children with ASD (random effects model).

For balance skills, 8 studies (n=182) reported the effects. Meta-analysis results (see **Figure 5**) showed a statistically significant pooled effect size (SMD = 0.85, 95% CI [0.54, 1.16], Z = 5.31, P < 0.001). This indicates that, compared to the control group, exercise interventions significantly improved balance skills in children with ASD, with a large effect size (SMD > 0.8). Heterogeneity testing indicated no heterogeneity among studies (I² = 0%, P = 0.51), suggesting that the positive effects found in different studies were highly consistent and the results robust.

**Figure 6:**
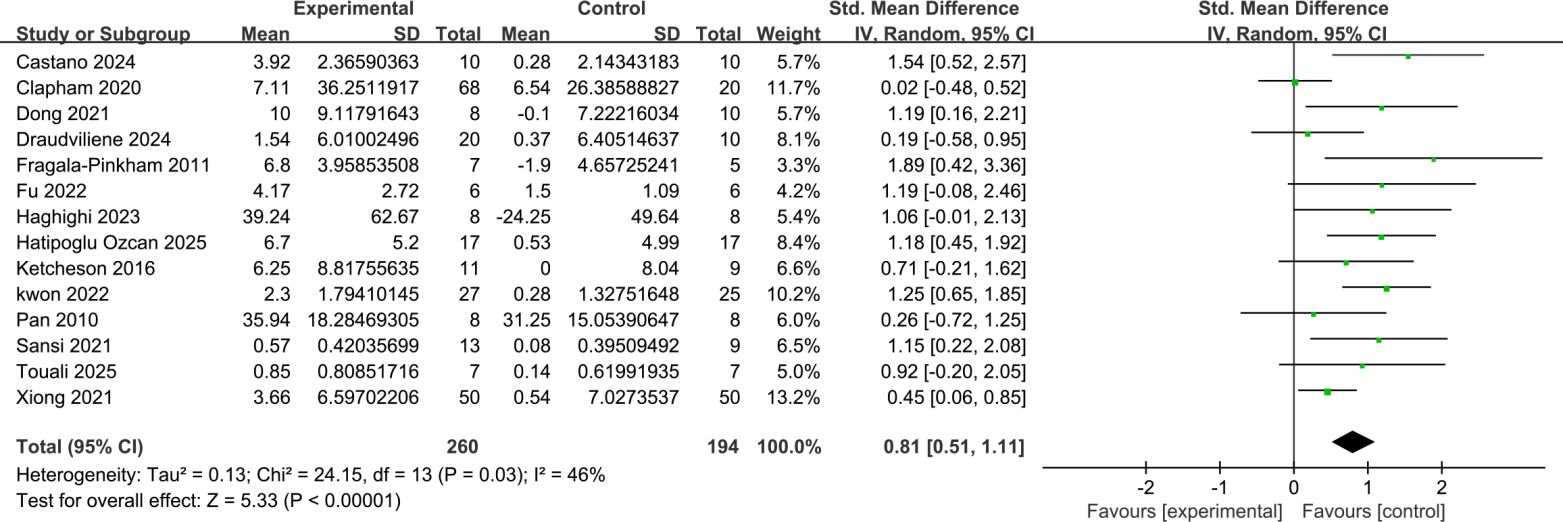
Forest plot of the effects of motor intervention on mobility skills in children with ASD (random effects model).

For locomotor skills, 14 studies (n=454) reported the effects on locomotor skills (e.g., running, jumping, sliding). The pooled effect size (see **Figure 6**) was statistically significant (SMD = 0.81, 95% CI [0.51, 1.11], Z = 5.33, P < 0.001), indicating a significant enhancing effect of exercise interventions on locomotor skills, with a large effect size (SMD > 0.8). Moderate heterogeneity was present among studies in this group (I² = 46%, P = 0.03), suggesting that differences in intervention protocols or participant characteristics across studies might influence the effect on locomotor skills.

#### 3.3.2 Subgroup analysis

This study further explored sources of heterogeneity through subgroup analysis.

When exercise type was used as the moderator variable, results showed that Land-based sports demonstrated significant improvements in all three skills (P < 0.001). However, their effect size confidence intervals partially overlapped with those of other exercise types, suggesting that absolute differences between exercise types should be interpreted cautiously. Aquatic sports only showed a significant improvement effect on locomotor skills (SMD = 0.93, P = 0.0001), while their effect on object control skills did not reach significance (P > 0.05). Notably, the number of studies in the Aquatic sports subgroup was small (n=2), potentially leading to insufficient statistical power; thus, the stability of these results requires further validation. Technology-assisted interventions showed no statistically significant effects on either locomotor or object control skills (P > 0.05).

When total intervention duration was used as the moderator variable, results showed that a total duration ≤ 1440 minutes produced significant improvements in balance, locomotor, and object control skills (P < 0.001). However, when the total duration was > 1440 minutes, the intervention regimen only showed a significant improvement effect on object control skills (SMD = 0.75, P = 0.003), while its effects on balance and locomotor skills did not reach statistical significance (P > 0.05). Details are shown in **Table 3**.

**Table 3:** Subgroup analysis of different exercise types and intervention durations on balance, locomotor, and object control dimensions.

Adjusting variable	Subgroups category	Meta-analysis results of balance			Meta-analysis results of movement			Meta-analysis results of control		
		SMD(95%CI)	Z	P	SMD(95%CI)	Z	P	SMD(95%CI)	Z	P
Motion Type	Land-based sports	0.85 [0.54, 1.16]	5.31	< 0.00001	0.94 [0.66, 1.23]	6.42	< 0.00001	0.83 [0.57, 1.09]	6.24	< 0.00001
	Aquatic sports	–	–	–	0.93 [0.45, 1.42]	3.81	0.0001	0.51 [-0.39, 1.41]	1.11	0.27
	Technical assistance Sports	–	–	–	0.56 [-0.11, 1.22]	1.64	0.1	0.75 [-0.30, 1.79]	1.4	0.16
Duration of intervention	≤1440min	0.67 [0.34, 1.00]	3.96	< 0.0001	0.90 [0.66, 1.14]	7.35	< 0.00001	0.85 [0.47, 1.23]	4.39	< 0.0001
	>1440min	0.88 [-0.11, 1.87]	1.74	0.08	0.62 [-0.10, 1.34]	1.69	0.09	0.75 [0.25, 1.25]	2.93	0.003

It is particularly important to note that in this study, the comparison of the effects among different types of exercises (land-based, water-based, and technology-assisted) was based on indirect subgroup analyses across different studies, rather than direct comparisons of subjects randomly assigned to different exercise types within the same study. Therefore, the interpretation of the differences in effects among different exercise types should be carried out with great caution, and the strength of the argument is lower than that of head-to-head randomized controlled trials.

#### 3.3.3 Publication bias and sensitivity analysis

Publication bias was comprehensively assessed using Begg’s test, funnel plots, trim-and-fill analysis, and sensitivity analysis (removing small-sample studies). The specific results are as follows:

For the balance skills subgroup, publication bias analysis was not performed due to the inclusion of only 8 studies. However, the results did not substantially change upon removal of individual studies or changing the model, suggesting some stability in the conclusion.

**Figure 7:**
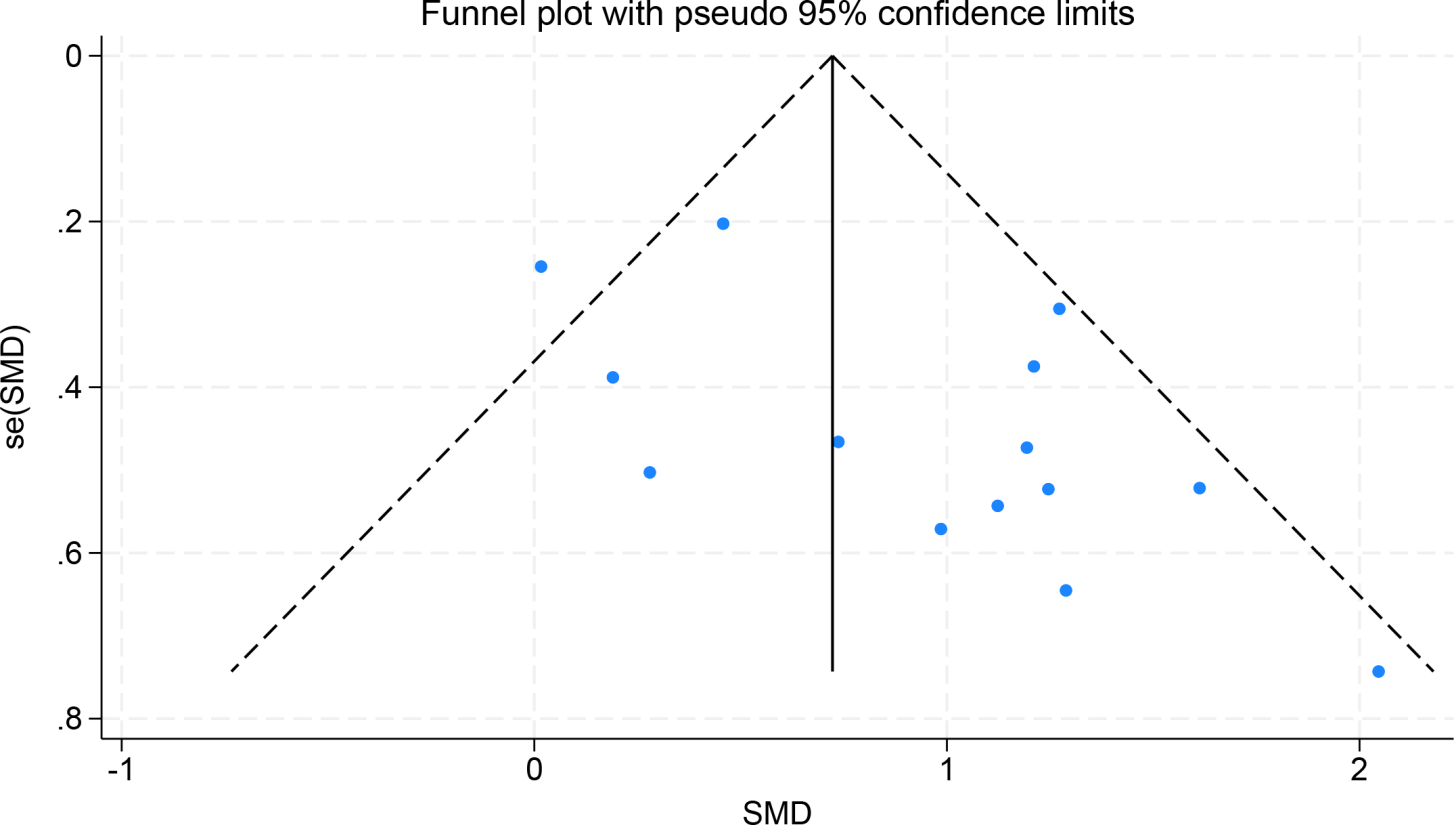
Publication bias funnel plot for evaluating the effect of exercise intervention on control skills in children with ASD.

For the object control skills subgroup, which included 14 studies, Begg’s test was performed using Hedges’s g as the effect size to analyze the correlation between effect size and standard error. The result showed z = 1.20, Prob > |z| = 0.2284 (P > 0.05), indicating no statistically significant publication bias. In the funnel plot, the study points were distributed symmetrically within the funnel, with no one-sided gap between large and small sample studies, further supporting the absence of publication bias in this subgroup. See **Figure 7**.

**Figure 8:**
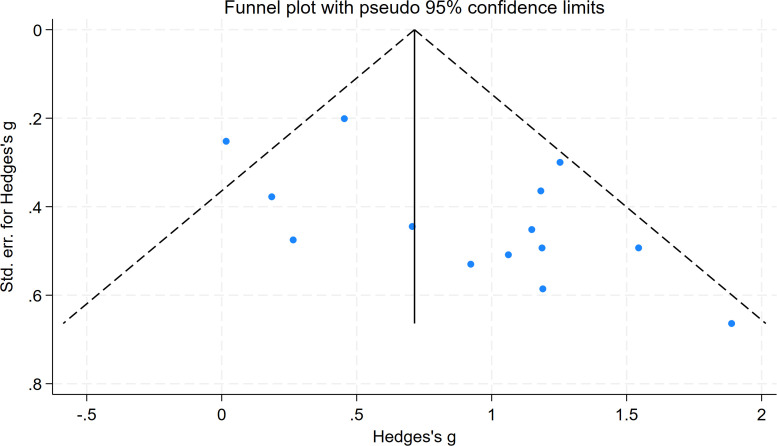
Publication bias funnel plot for evaluating the effect of motor intervention on the mobility skills of children with ASD.

**Figure 9:**
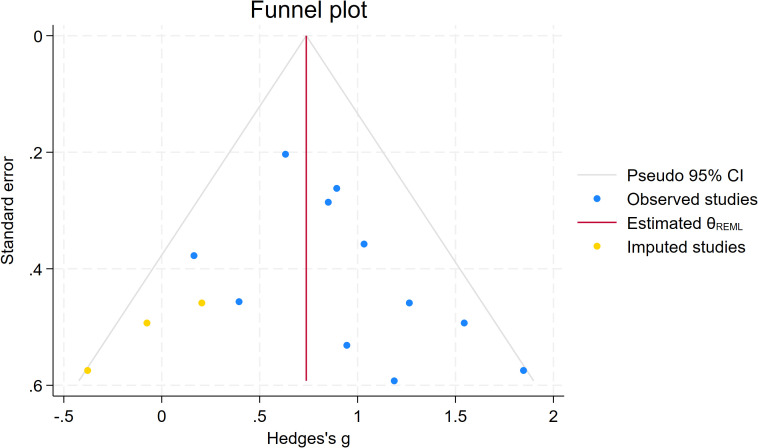
Funnel plot of the effect of motor intervention corrected by the clipping method on the mobility skills of children with ASD.

For the locomotor skills subgroup, which included 13 studies, the result showed z = 2.38, Prob > |z| = 0.0173 (P < 0.05), indicating statistically significant publication bias. The funnel plot showed potential asymmetry in the distribution of study points, suggesting that small-sample studies with non-significant effects might not have been fully included. The trim-and-fill analysis filled hypothetical unpublished studies (yellow points), visually presenting the potential impact of publication bias and indicating the need for further verification of result stability. See **Figures 8** and **9**.

After removing small-sample studies from the locomotor skills subgroup, Begg’s test was performed again. The result showed z = 1.87, Prob > |z| = 0.0617 (P > 0.05), and the statistical significance of publication bias disappeared. This indicates that the initial publication bias in the original subgroup was primarily driven by small-sample studies, and the result stability significantly improved after their removal.

In summary, the object control skills subgroup showed no statistically significant publication bias; the locomotor skills subgroup initially showed publication bias, but after sensitivity analysis by “removing small-sample studies,” the statistical significance of the bias disappeared, and the results showed good stability.

## 4 Discussion

### 4.1 Main findings

Through subgroup analysis, this study systematically compared, for the first time, the effects of different exercise types and intervention doses on the three core dimensions of gross motor skills in children with ASD. The meta-analysis, synthesizing data from 16 studies involving 493 participants, indicates that exercise interventions have a significant and positive overall effect on improving gross motor skills in children with ASD (SMD = 0.81–0.86), providing key evidence for exercise as an effective supplementary rehabilitation approach for children with ASD (28).

The core value of this study lies in revealing heterogeneity of effects through subgroup analysis. Exercise type is a key factor contributing to heterogeneity. Land-based sports demonstrated the most consistent and largest positive effects across all three skill dimensions, making them the most recommendable form of exercise intervention in the current evidence base. In contrast, Aquatic sports only showed significant improvement in locomotor skills, while technology-assisted interventions (e.g., VR training) did not show stable significant benefits in this analysis.

Regarding intervention dose, this study found a non-linear dose-response relationship. The medium-dose regimen with a total duration ≤ 1440 minutes (approx. 8–12 weeks) produced significant improvements in balance, locomotor, and object control skills, suggesting this range may be the “efficient window period” for generating broad benefits. Although the high-dose regimen (> 1440 minutes) maintained a significant improvement in object control skills, its effects on balance and locomotor skills did not reach statistical significance. This may be related to the small number of studies and sample size in the high-dose group rather than fully reflecting dose ineffectiveness.

The above comparison of different types of exercises is based on subgroup analysis across studies. This indirect comparison across studies is significantly less powerful than a direct randomized controlled comparison. Therefore, the current conclusions regarding the differences in the effects of different types of exercises should be interpreted with caution. Future verification of these differences is needed through head-to-head randomized controlled trials.

### 4.2 Exploration of mechanisms

#### 4.2.1 Effect differences between exercise types

Most of the land-based exercises focus on structured and repetitive skill training, which can directly target the core symptoms of ASD children, such as body coordination, balance, mobility, and object control (44). Some studies have mentioned that such training may improve motor performance by promoting sensory integration and neural plasticity, but these potential mechanisms were not directly measured in this included study. Therefore, further verification is needed in future research by combining neuroimaging evidence (45, 46). In terms of skill specificity, an important methodological consideration is “content matching”: most of the assessment tools included in this study (such as TGMD, BOT-2, BOTMP) have test items mainly focused on land-based movement skills. Therefore, the high degree of match between land-based movement intervention and assessment content may contribute to its large observed effect size to some extent. This suggests that the current measurement tools may systematically bias towards capturing the benefits of land-based movement (47). Future research developing or adopting assessment tools with greater ecological validity or environmental universality will help to more fairly compare the effects of different exercise patterns.

Technology-assisted training holds potential but also harbors non-negligible limitations (48–50). Technologies like VR can provide immediate feedback and highly structured environments, which to some extent attract and help reduce anxiety in children with ASD (51). However, their effects were smaller, possibly because children with ASD have significant deficits in using sensory feedback to control movement. VR tasks involve using visual feedback to sequence simple tasks and interact with objects in 3D space, and the generalization of skills from virtual to real-world environments is challenging. Therefore, VR technology needs to be combined with other motor development activities (52–54).

The less pronounced effects of Aquatic sports may be because water buoyancy reduces weight-bearing and joint stress, which may simultaneously lower the demands on muscle strength and postural control (55). For children with ASD whose primary goals are to improve strength, coordination, and balance, the aquatic environment may be less challenging than land-based training, leading to poorer skill transfer (44).

#### 4.2.2 Dose-response relationship

In examining the dose-response relationship of the total intervention dose, our findings indicate that the medium-dose regimen (total duration ≤ 1440 minutes) aligns most closely with the established principles of motor skill acquisition and demonstrates broad effectiveness. This dosage range, typically corresponding to a conventional 8- to 12-week training cycle, provides sufficient cumulative practice time. This allows children with ASD to move past an initial period of adjustment to novel activities and facilitates the critical transition of motor skills from the cognitive to the associative stage of learning, resulting in significant synchronous improvement across multiple gross motor skill domains (56).

In contrast, while the high-dose regimen (total duration > 1440 minutes) may potentially yield more stable or sustained benefits, its effects on improving balance and locomotor skills did not reach statistical significance in our analysis (57). This observation may be attributable to several factors. First, overly extended intervention cycles might lead to a natural decline in participant motivation over time, thereby affecting engagement and adherence. Second, certain fundamental motor skills may reach a performance plateau relatively early, leading to diminishing marginal returns from continued intervention (58). Additionally, in longer-term studies, the developmental progress observed in control group children due to natural maturation may partially dilute the apparent net benefit attributable to the intervention (59).

### 4.3 Limitations

Although this study conducted a systematic review and Meta-analysis of the included literature, it still has the following limitations. Firstly, in the research methods section, some of the included studies were non-randomized controlled trials, and there was a high risk of bias in aspects such as the generation of randomization sequences, the concealment of allocation, and the implementation of blinding for subjects and intervention implementers (60). These methodological limitations may have overestimated the overall intervention effect to some extent. Secondly, in terms of outcome measurement, as mentioned earlier, the mainstream gross motor assessment tools used in this study (such as TGMD, BOT-2) mainly target motor skills in the terrestrial environment. This measurement characteristic may give a natural advantage to terrestrial movement interventions, while failing to fully capture the unique benefits of water-based or technology-assisted movements, thereby affecting the fairness of comparisons between different types of movements. Thirdly, in the analysis section, the intervention dose threshold set in this study (1440 minutes) was pre-defined based on the distribution of existing studies and clinical experience, but it still has a certain degree of subjectivity and exploratory nature. Its universality needs to be further verified in different populations and environments. Although subgroup analysis was conducted based on movement type and total dose, and lower heterogeneity was observed in some results (such as balance and manipulation skills), there was still moderate heterogeneity in the displacement skill results (I² = 46%), indicating that there are other potential influencing factors, such as the age of the subjects, the severity of symptoms, family participation, and the intervention implementation environment, which have not been adequately controlled. Fourthly, in the evidence base section, in some subgroups, such as water-based movements, technology-assisted movements, and high-dose intervention groups, the number of included studies was limited, resulting in low statistical power and insufficient stability of the corresponding results. The conclusions need to be verified by more high-quality studies. Fifthly, the included studies lacked long-term follow-up data, making it impossible to assess the persistence of the movement effects. Finally, although a publication bias test was conducted, the asymmetry of the funnel plot suggests the possibility of unpublished negative results, which may overestimate the overall effect size.

### 4.4 Implications and future directions

Based on the findings of this study, structured land-based exercise interventions should be prioritized in future clinical rehabilitation and special education practices, as they have been demonstrated to significantly and comprehensively enhance balance, locomotor, and object control skills in children with ASD. Concerning intervention dosage, it is recommended to limit the total duration to within 1440 minutes, as this range has been identified as a key window for achieving optimal benefits. For Aquatic sports and technology-assisted training, current evidence does not support their widespread adoption as core intervention programs. However, they may be considered as personalized supplementary approaches when resources permit, provided that attention is given to program standardization and individual adaptability (61).

Regarding future research directions, it is recommended to conduct additional high-quality, large-sample randomized controlled trials. Efforts should focus on strengthening the evidence base in areas currently supported by limited data, such as Aquatic sports and technology-assisted interventions. Furthermore, research should further explore the adaptive relationship between different intervention dosages and variables such as child age and symptom severity, to identify the “optimal dose” for specific populations. The incorporation of neuroimaging techniques, such as fMRI and fNIRS, is encouraged to elucidate the neural mechanisms underlying the improvement of gross motor skills through exercise interventions (62). Additionally, future studies are advised to employ prospective trial registration, use standardized gross motor assessment tools, and actively publish negative or neutral findings to enhance the transparency and comparability of the evidence.

## 5 Conclusion

The findings indicate that exercise intervention is an effective means of improving gross motor skills in children with ASD. Among them, structured Land-based sports demonstrate the most robust and comprehensive promotive effects, simultaneously producing large, positive impacts on the balance, locomotor, and object control skills of children with ASD. Regarding intervention dosage, a medium-dosage regimen with a total duration controlled within 1440 minutes has been confirmed as the key window for optimal benefits. The effects of different exercise types vary significantly, suggesting that in practice, interventions with sufficient evidence and clear benefits should be prioritized and personalized based on individual characteristics and rehabilitation goals. This study provides important evidence-based grounds for integrating scientific and systematic exercise interventions into the comprehensive rehabilitation and educational support system for children with ASD.

## Data Availability

The original contributions presented in the study are included in the article/**Supplementary Material**. Further inquiries can be directed to the corresponding author.
