# Supplementary material for: Effectiveness of exercise interventions on gross motor skills in children with autism spectrum disorder: a systematic review and meta-analysis
Source: Front Psychiatry. 2026 Feb 26;17:1745638. doi: 10.3389/fpsyt.2026.1745638 (PMC12980882; doi:10.3389/fpsyt.2026.1745638)
Supplement: Supplementary file 2 [file Table2.docx]

| Adjusting variable | Subgroups category | Meta-analysis results of balance | | | Meta-analysis results  of movement | | | Meta-analysis results  of control | | |
| --- | --- | --- | --- | --- | --- | --- | --- | --- | --- | --- |
|  |  | SMD(95%CI) | Z | P | SMD(95%CI) | Z | P | SMD(95%CI) | Z | P |
| Motion Type | Land Sports | 0.85 [0.54, 1.16] | 5.31 | < 0.00001 | 0.94 [0.66, 1.23] | 6.42 | < 0.00001 | 0.83 [0.57, 1.09] | 6.24 | < 0.00001 |
|  | Water Sports | - | - | - | 0.93 [0.45, 1.42] | 3.81 | 0.0001 | 0.51 [-0.39, 1.41] | 1.11 | 0.27 |
|  | Technical assistance Sports | - | - | - | 0.56 [-0.11, 1.22] | 1.64 | 0.1 | 0.75 [-0.30, 1.79] | 1.4 | 0.16 |
| Duration of intervention | ≤1440min | 0.67 [0.34, 1.00] | 3.96 | < 0.0001 | 0.90 [0.66, 1.14] | 7.35 | < 0.00001 | 0.85 [0.47, 1.23] | 4.39 | < 0.0001 |
|  | ＞1440min | 0.88 [-0.11, 1.87] | 1.74 | 0.08 | 0.62 [-0.10, 1.34] | 1.69 | 0.09 | 0.75 [0.25, 1.25] | 2.93 | 0.003 |

| Study | Country | Diagnostic | Intervention | Study Design | E/C | Total minute | Week | Frequency | Single duration | Outcome indicator |
| --- | --- | --- | --- | --- | --- | --- | --- | --- | --- | --- |
| Bremer 2014 | Canada | DSM-V | Land Sports | WLCD | 5/4 | 1440 | 12 | 2 | 60 | PDMS-2 |
| Castaño 2024 | Columbia | DSM-V | Land Sports | RCT | 10/10 | 1440 | 8 | 3 | 60 | ADS-3 |
| Clapham 2020 | America | DSM-V | Water Sports | CCD | 71/20 | 960 | 8 | 2 | 60 | BOT-2 |
| Dong 2021 | China | DSM-V、ADI-R、C-PEP3 | Land Sports | RCT | 8/10 | 1800 | 10 | 3 | 60 | TGMD-3 |
| Draudvilienė 2024 | Republic of Lithuania | DSM-V | Technical assistance sports | RCT | 10/10 | 450 | 5 | 3 | 30 | BOTMP |
| Fragala-Pinkham 2011 | America | DSM-V | Water Sports | WLCD | 7/5 | 1120 | 14 | 2 | 40 | YMCA、M-PEDI |
| Fu 2022 | China | DSM-V | Land Sports | RCT | 6/6 | 1440 | 8 | 3 | 60 | TGMD-2 |
| Haghighi 2023 | Iran | GARS-2、WISC-4 | Land Sports | RCT | 8/8 | 1440 | 8 | 3 | 60 | GARS-2 |
| Hatipoğlu Özcan 2025 | Turkey | DSM-V、GARS-2-TV | Land Sports | RCT | 17/17 | 1440 | 12 | 2 | 60 | PMDS-2 |
| Ketcheson 2016 | America | DSM-IV、ADOS-2 | Land Sports | QED | 11/9 | 9600 | 8 | 5 | 240 | TGMD-2 |
| Kwon 2022 | Korea | DSM-V | Technical assistance sports | RCT | 27/25 | 1080 | 12 | 2 | 30 | TGMD-3 |
| Najafabadi 2018 | Iran | DSM-IV-TR、GARS-2 | Land Sports | QED | 14/14 | 1440 | 12 | 3 | 40 | BOTMP |
| Pan 2010 | Taiwan, China | DSM-IV | Water Sports | COD | 8/8 | 1680 | 12 | 2 | 70 | HAAR |
| Sansi 2021 | Turkey | DSM-V | Land Sports | MMR | 13/18 | 1440 | 12 | 2 | 60 | BOT-2 |
| Touali 2025 | Morocco | DSM-V | Land Sports | QED | 7/7 | 2025 | 15 | 3 | 45 | UQAC-UQAM |
| Xiong 2021 | China | DSM-V | Land Sports | RCT | 50/50 | 1200 | 10 | 2 | 60 | ABC |

|  |
| --- |

| Serial number | Search content |
| --- | --- |
| 1 | "Autism Spectrum Disorder"[Mesh] OR "Autistic Disorder"[Mesh] OR autism OR autistic OR "ASD" OR asperger* OR "pervasive developmental disorder" |
| 2 | "Exercise"[Mesh] OR "Exercise Therapy"[Mesh] OR "Motor Activity"[Mesh] OR "Sports"[Mesh] OR "physical activit" OR "physical training" OR "motor intervention" OR "exercise intervention" OR "movement intervention" OR sport |
| 3 | "Motor Skills"[Mesh] OR "motor skill*" OR "motor competence" OR "gross motor" OR "locomotor skill" OR "object control" OR "movement proficiency" |
| 4 | "Child"[Mesh] OR "Child, Preschool"[Mesh] OR child* OR preschool* OR kid OR kids OR toddler* OR boy OR boys OR girl OR girls |
| 5 | #1 AND #2 AND #3 AND #4 |
